# Effect of sorghum-based diets on the abundance and diversity of ruminal methanogenic archaea in Mashona goats

**DOI:** 10.3389/fmicb.2026.1762242

**Published:** 2026-04-01

**Authors:** Nyasha Rugwete, Tonderai Mutibvu, Elijah Nyakudya, Khanyisile Hadebe, Tinyiko Edward Halimani

**Affiliations:** 1 Department of Research and Specialist Services, Harare, Zimbabwe; 2 Department of Livestock Sciences, University of Zimbabwe, Harare, Zimbabwe; 3 Agricultural Research Council Onderstepoort Veterinary Research, Pretoria, South Africa

**Keywords:** archaea, indigenous goats, meta-taxonomics, methane emission, sorghum

## Abstract

Dietary manipulation offers an effective strategy for modifying rumen microbiota to reduce methane emissions. The study assessed the influence of sorghum-based diets on the ruminal methanogenic archaeal community in Mashona goats. The diets were formulated as follows; PS:50-50% (plain or untreated sorghum); SS:75-75% (soaked sorghum at 75% inclusion); FS:25-25% (fermented sorghum at 25% inclusion); MS:100-100% (malted sorghum at 100% inclusion); M:100-(100% maize-based) and CC-a commercial concentrate as the control. A total of 48 Mashona goats blocked by sex were randomly allocated into six treatments of eight animals each in a randomized block design (RBD). Selection of experimental animals was based on breed characteristics. The selected goats had an initial age range of approximately 12 months and an average initial body weight of 16–17 kg. A total of 16 rumen samples were collected from the experimental animals after a 15-week feeding trial for DNA extraction and microbiota analysis. Results obtained revealed that diet significantly influenced archaeal composition, with the phylum Euryarchaeota and genus *Methanobrevibacter* dominating across all dietary treatments. The FS:25 diet exhibited a 99.4% dominance of Euryarchaeota while the MS:100 diet had a representation of 55% Euryarchaeota and 45% Candidatus Thermoplasmatota. The phylum Candidatus Thermoplasmatota represented by genus *Methanomethylophilus* was observed in low abundance across treatments. While diet did not significantly influence microbial diversity (*p* > 0.05), a numerical trend was observed, with the MS:100 diet showing the highest Shannon diversity index (1.66 ± 0.11) and the commercial concentrate exhibiting the lowest (1.13 ± 0.40) numerical diversity. The study concluded that the methanogenic archaea responded differently to every dietary treatment. Notably, the Bray–Curtis PCoA identified FS:25 and CC diets as distinct outliers with *Methanosarcina* and *Methanomicrobium* exclusively observed in FS:25 and CC, respectively. Future research could be designed toward *in vivo* methane emission quantification for the MS:100 diet to confirm its mitigation potential and optimal dietary status. The diversity in the methanogenic community in MS:100 reflects an integrated rumen ecosystem function. Direct quantification of enteric methane emissions through *in vivo* measurements is necessary to validate whether this favorable microbial balance translates into measurable reductions in methane output.

## Introduction

The rumen is an intricate system comprising diverse microorganisms such as bacteria, archaea, ciliate protozoa, fungi, as well as viruses (Matthews et al., 2019; Zhu et al., 2022). The link between an animal’s diet and the ruminal microbiome is vital in maintaining its overall health and productivity. Diet can affect microbiota dynamics such as diversity and populations (Newbold and Ramos-Morales, 2020). When appropriately formulated, sorghum-based diets can stimulate the development of a healthy and balanced rumen environment (Palmonari et al., 2024). Several dietary factors such as fiber, tannin content and starch structure can critically influence the composition and activity of rumen microbial populations, particularly methanogenic archaea (Mcallister et al., 2025). Fiber composition plays a fundamental role in shaping microbial communities, as structural carbohydrates such as cellulose and hemicellulose promote fibrolytic bacteria that produce a range of fermentation end-products (Piao et al., 2014).

Feed processing methods such as malting, soaking and fermentation can substantially alter sorghum grain characteristics and their impact on rumen fermentation and archaea populations. Malting activates endogenous enzymes that modify starch structure and increase the availability of fermentable substrates, potentially altering the balance between hydrogen production and utilization (Nkhata et al., 2018). Soaking hydrates grains and initiates enzymatic activity, affecting starch gelatinization and accessibility to microbial degradation. Fermentation of grain employs microbial activity to pre-digest nutrients and generate organic acids and other metabolites that can influence rumen pH and microbial population dynamics (Guzmán-ortiz et al., 2018). These processing techniques can modify the tannin content, starch digestibility, and fiber characteristics of sorghum, consequently affecting methanogenic archaeal populations through multiple mechanisms (Ojha et al., 2018).

The rumen function which is key to ruminant productivity can also be regulated through diet and management (Liu et al., 2021). As the global population continues to rise, the demand for meat production increases, necessitating enhanced productivity. Understanding the rumen archaeal community composition and its response to dietary interventions is foundational for developing feeding strategies that could potentially reduce energy loses through methane production (Hassan F. U. et al., 2020; Hassan S. et al., 2020). Methanogenesis by archaea results in a 2–12% loss of gross energy intake (Patra et al., 2017). Characterizing rumen microbial populations through feed management represents an emerging approach for identifying dietary formulations that may influence methane-producing pathways (Króliczewska et al., 2023). Sorghum-based diets have shown potential, as the higher tannin content and slower ruminal fermentation rate of sorghum, relative to maize, may modulate archaeal activity and partially redirect fermentation energy away from methanogenic pathways, potentially reducing the proportion of gross energy lost as methane. These strategies have potential to mitigate adverse environmental impacts while simultaneously enhancing production efficiency, though their effects on actual methane emissions require validation through direct measurement (Matthews et al., 2019). Further research, including *in vivo* methane quantification, is required to validate and assess the impact of microbiome shifts on reducing methane emission.

Dietary interventions, such as sorghum-based diets, can significantly influence rumen archaeal communities. Mashona goats, indigenous to Zimbabwe and other Southern African countries are particularly well-suited for investigating such interventions, as they are adapted to seasonal feed scarcity and variable forage quality. These adaptive traits, combined with their predominant management under extensive or semi-intensive suggest that their rumen archaeal communities may respond distinctively to sorghum-based diets. Understanding these responses is therefore of direct practical relevance for smallholder farming contexts across Southern Africa, particularly in the semi-arid and arid regions where goat production is prevalent. Such knowledge can inform targeted supplementation strategies that leverage sorghum as a climate-smart feed ingredient, ultimately enhancing the productivity and sustainability of Mashona goat production systems. The effects of sorghum on the populations and diversity of archaeal communities in the goat rumen have not been extensively researched, highlighting a critical area for further investigation to optimize both productivity and sustainability in goat farming. There is a scarcity of information on the influence of sorghum grain-based diets on the diversity and abundance of goat microbiota. The purpose of the study was, therefore, characterize the archaeal community composition and diversity in the goat rumen in response to sorghum-based diets on the archaeal population in the goat rumen, exploring how sorghum can be effectively utilized as a climate-smart dietary ingredient for goats. It was hypothesized that sorghum-based diets would alter the diversity and community composition of ruminal methanogenic archaea in Mashona goats, reducing the abundance of dominant methanogens.

## Materials and methods

A meta-taxonomic community analysis focusing on methanogenic archaea was conducted to provide a detailed characterization of the rumen microbiota, identifying crucial methane-producing microbes and their response to different diets.

### Animal ethics

Animal handling and welfare was conducted following guidelines by the National Animal Research Ethics Committee Statutory Instrument 246 of 2021 (National Animal Research Ethics Committe, 2021) and internationally recognized standards for research animal welfare. Zimbabwe National Animal Research Ethics Committee approved the research trial (Reference number 008/23).

### Sorghum processing

The unprocessed grains for the control treatment were cleaned and subjected to nutritional and phytochemical analyses. For the soaking treatment, grains were washed with tap water and steeped in water for 48 h at room temperature as described by Bertin and Carly (2023) at a grain to water ratio of 1:5 (w/v). The soaking procedure involved replacing the water every 12 h to ensure optimal grain hydration while preventing microbial growth and fermentation. Each water replacement was followed by a 1-h air rest to promote uniform water absorption, thereby enhancing moisture content in the grain (Turner et al., 2019). After steeping, grains were dried in an oven at 50 °C for a 24-h period. The steeping procedure for the malted grains is as described for the soaking treatment. After steeping, the grains were placed in sterilized wet hessian and stored in a dark room to sprout at room temperature for 48 h. The fermented grains were cleaned and steeped in water for 5 days in an airtight container to ferment at room temperature at a grain to water ratio of 1:5 (w/v). Grains were dried at 50 °C for 24 h post fermentation.

### Animal and feed management

A total of 48 Mashona goats blocked by sex were randomly divided into six treatments of eight animals each in a randomized block design (RBD). The goats were selected for their breed characteristics, with an initial age range of approximately 12 months and an average initial body weight of 16–17 kg. The sorghum-based diets selected for the feeding trial were as follows; PS:50; SS:75; MS:100 and FS:25 formulated from plain sorghum at 50% inclusion, soaked sorghum at 75% inclusion, malted sorghum at 100% inclusion, and fermented sorghum at 25% inclusion, respectively. A maize-based diet M:100 formulated from 100% maize and a commercial maize-based concentrate feed (CC) were the control diets. Animals were placed in individual cages and each animal received 200 g of the experimental diet daily, with hay and water provided *ad libitum*. The nutritional composition of the feed is shown in Table 1.

**Table 1:** Chemical composition of formulated diets.

Variables %							
Diet	DM	Ash	EE	ADF	NDF	CP	ME (MJ/kg)
PS:50	91.84	4.85	7.03	9.57	27.39	10.59	10.71
SS:75	90.99	4.69	7.82	7.80	19.56	11.34	11.13
MS:100	92.17	4.88	5.95	10.69	22.80	11.48	10.71
FS:25	92.23	4.53	4.99	10.98	25.04	10.75	10.34
M:100	90.71	5.14	6.65	11.03	29.66	10.89	10.38
CC	92.40	5.55	3.64	12.99	28.85	10.41	9.50
Hay	95.54	5.42	2.27	67.76	79.45	6.63	4.50

PS:50, 50% plain sorghum diet; SS:75, 75% soaked sorghum diet; MS:100, 100% malted sorghum diet; FS:25, 25% fermented sorghum diet, M:100, 100% maize-based diet; CC, commercial concentrate; Hay, veld hay; DM, dry matter; EE, ether extract; ADF, acid detergent fiber; NDF, neutral detergent fiber; CP, crude protein.

### Rumen sampling

After 15 weeks of the feeding trial, the goats were moved to a registered abattoir located in Harare, 52 km from the experimental site. The animals were fasted for 24 h before slaughter. To ensure humane treatment during transportation, the goats were loaded into well-ventilated vehicles designed for livestock, minimizing stress and discomfort. The journey was conducted at a moderate pace to reduce agitation, and the animals were monitored closely for signs of distress. Humane slaughter and dressing were conducted in line with commercial protocols at a registered abattoir. Immediately upon slaughter, rumen digesta samples were collected directly from the rumen using sterile collection procedures to prevent contamination. Approximately 50 g of whole rumen contents (including both liquid and solid fractions) were harvested from the central ventral sac of the rumen from each animal. Samples were collected within 15 min post-slaughter to minimize microbial population changes associated with post-mortem conditions. The collected rumen digesta was immediately placed into sterile 50 mL conical tubes and snap-frozen in liquid nitrogen to preserve microbial DNA integrity and prevent RNA degradation. Samples were transported to the laboratory and stored at −80 °C until DNA extraction to maintain sample integrity and minimize microbial population shifts during storage. Standard methodologies (Aphale and Kulkarni, 2018) were applied to partition the digesta into liquid, fiber-adherent, and fiber-associated microbe fractions, ensuring comprehensive representation of the archaeal communities across different rumen niches. The liquid fraction was obtained by straining the thawed rumen content through four layers of sterile cheesecloth. The fiber-adherent fraction was collected from material retained on the cheesecloth and obtained by washing the fibrous material with sterile phosphate-buffered saline (PBS). These fractions were then combined for subsequent metagenomic DNA extraction.

### Sample collection and DNA extraction and sequencing

Rumen samples were collected from three animals per treatment for DNA extraction and downstream sequencing analysis, yielding an initial total of 18 samples. However, two samples were lost during laboratory processing, resulting in a final dataset of 16 samples available for analysis. The distribution of biological replicates per treatment was as follows: PS:50 (*n* = 3), SS:75 (*n* = 3), FS:25 (*n* = 3), MS:100 (*n* = 2), M:100 (*n* = 2), and CC (*n* = 3). The Invitrogen DNA isolation kit (Invitrogen^™^ K182104A) was used to extract microbial genetic material from rumen samples, following the standard procedures outlined by the manufacturer. To assess the quality and quantity of the extracted DNA, gel electrophoresis was performed alongside spectrophotometric analysis. Gel electrophoresis was used to verify the integrity of the DNA, ensuring that no degradation occurred during the extraction process. A Nanodrop 2000 spectrophotometer (Thermo Scientific, Madison, United States) was used to quantify the extracted DNA, allowing for precise measurement of DNA concentration and purity ratios. The archaeal communities were investigated through PCR amplification of the V4 region of the 16S rRNA gene. The following primers were used for amplification: Ar915aF (5′-AGG AAT TGG CGG GGG GAG C-3′) and Ar1386R (5′-GCG GTT GGT GTC GCA AGG A-3′). The PCR conditions for archaeal amplification included an initial denaturation step at 95 °C for 5 min, followed by 30 cycles of denaturation at 95 °C for 20 s, annealing at 55 °C for 15 s, and extension at 72 °C for 5 min, concluding with a final extension at 72 °C for 10 min. Amplified DNA products were then purified and sequenced on an Illumina MiSeq platform (Illumina, California, United States). Raw fastq files were obtained from the Illumina Miseq.

### Bioinformatics

Quality evaluation of the initial sequencing data was conducted using FastQC version 0.12.1, focusing on adapter contamination, GC content distribution, per-base Phred scores, and sequence duplication rates. Raw sequencing data from 16 samples exhibited satisfactory initial quality and consistent 150 bp read lengths prior to processing. Fastp v0.23.4 software was used to trim adapter sequences and perform quality-based filtration. Following Trimmomatic processing, low-quality bases (*Q* < 20) and readings <50 bp were eliminated, retaining 85% of the raw data (8.5 million/sample). The read length distribution transitioned from a pre-trim homogeneity of 150 bp to a post-trim profile of 100–140 bp, indicating successful adaptor removal. Taxonomy classification was performed using Kraken2 v2.1.2 at a confidence level of 0.1, utilizing the Standard-64GB database (RefSeq 2023-10 release). The Kraken2/Bracken pipeline was chosen due to its use of the comprehensive, genome-based RefSeq database. This database provides better taxonomic resolution for rumen archaea compared to 16S rRNA-specific databases. Additionally, Kraken2/Bracken offers increased speed and accuracy in classifying microbial sequences within complex communities (Stewart et al., 2019). Optimal sensitivity for detecting low-abundance methanogenic species was achieved with a confidence threshold of 0.1. To reduce the possibility of false-positive results, the abundances were tweaked using Bracken v 2.8. Only taxa with a relative abundance of 0.1% or higher in one sample were kept for further analysis. This cautious filtering method has been shown to be effective in previous metagenomic research (Beaulaurier et al., 2021). Species-level abundance was re-evaluated with Bracken v2.8. The dominance of phyla and genera was visualized using Hierarchical Krona charts and Sankey diagrams (R package: ggsankey). Alpha diversity was assessed with the Shannon index based on rarefied data (using Kruskal–Wallis tests for significance). Methanogen community composition/beta diversity was compared via principal coordinate analysis (PCoA), utilizing Bray–Curtis dissimilarity.

### Statistical analyses

One-way analysis of variance (ANOVA) and Duncan’s multiple range tests were conducted in SAS (version 9.4) to assess ruminal methanogen diversity indices and the composition of the methanogen community at both phylum and genus levels. Differences were considered statistically significant at *p* < 0.05.

## Results

### Taxonomic composition and diversity

**Figure 1:**
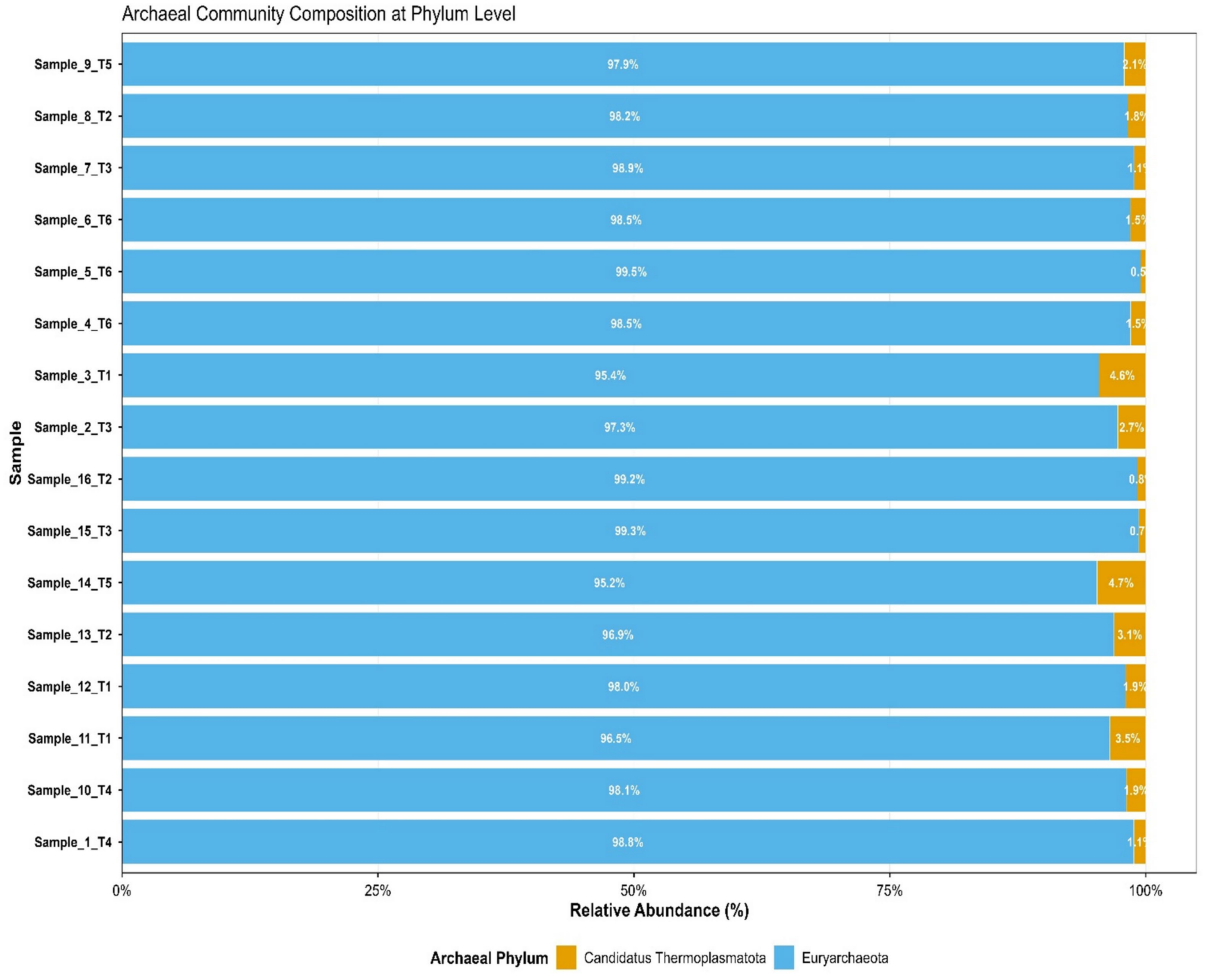
Stacked bar plot of archaeal relative abundance by treatment. T1–T6: treatment 1–treatment 6; T1-PS:50; T2-SS:75; T3-FS:25; T4-MS:100; T5-M:100; T6-CC-commercial concentrate.

**Figure 2:**
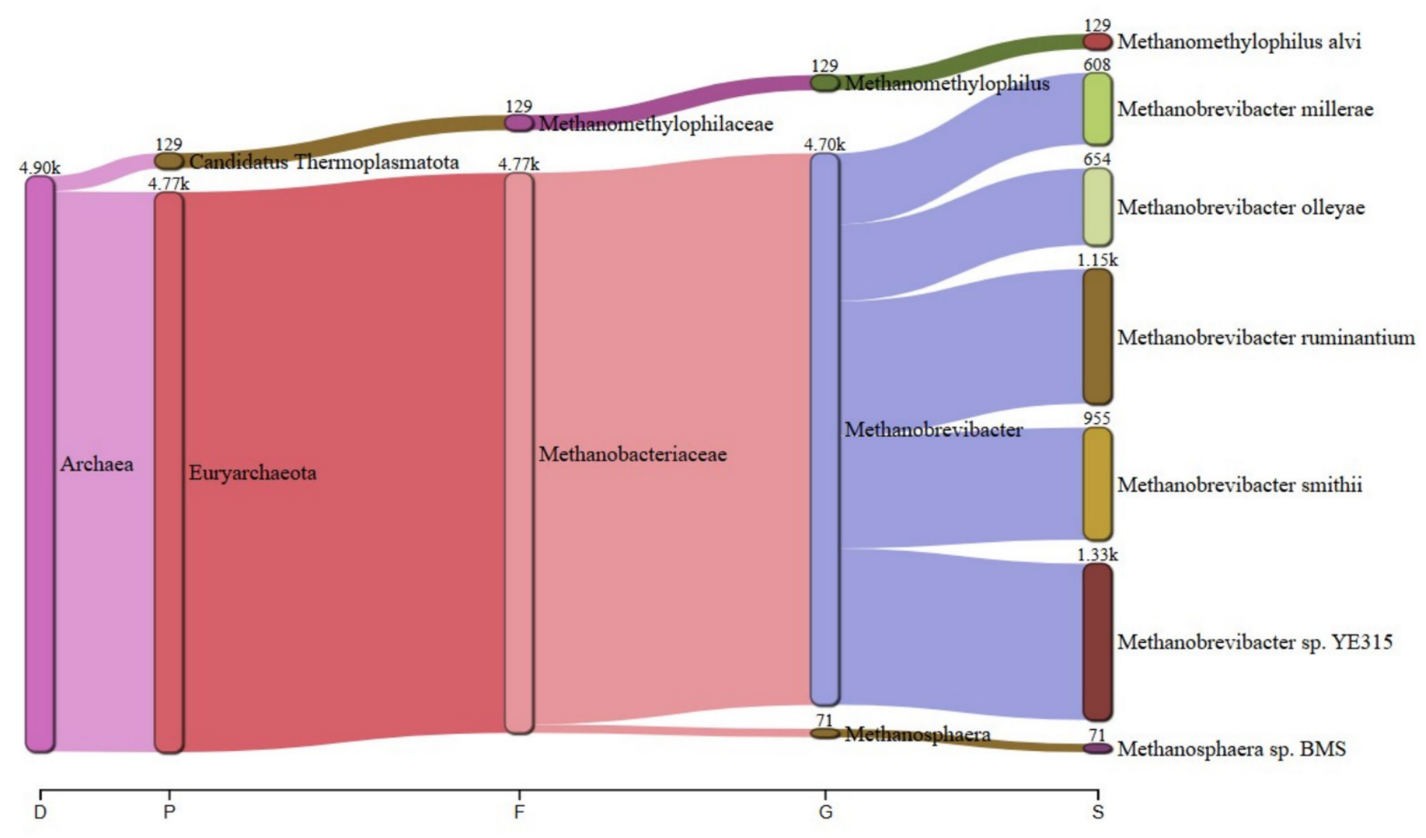
Treatment-specific taxonomic composition of treatment PS:50 dominated by *Methanobrevibacter* sp. *YE315*.

**Figure 3:**
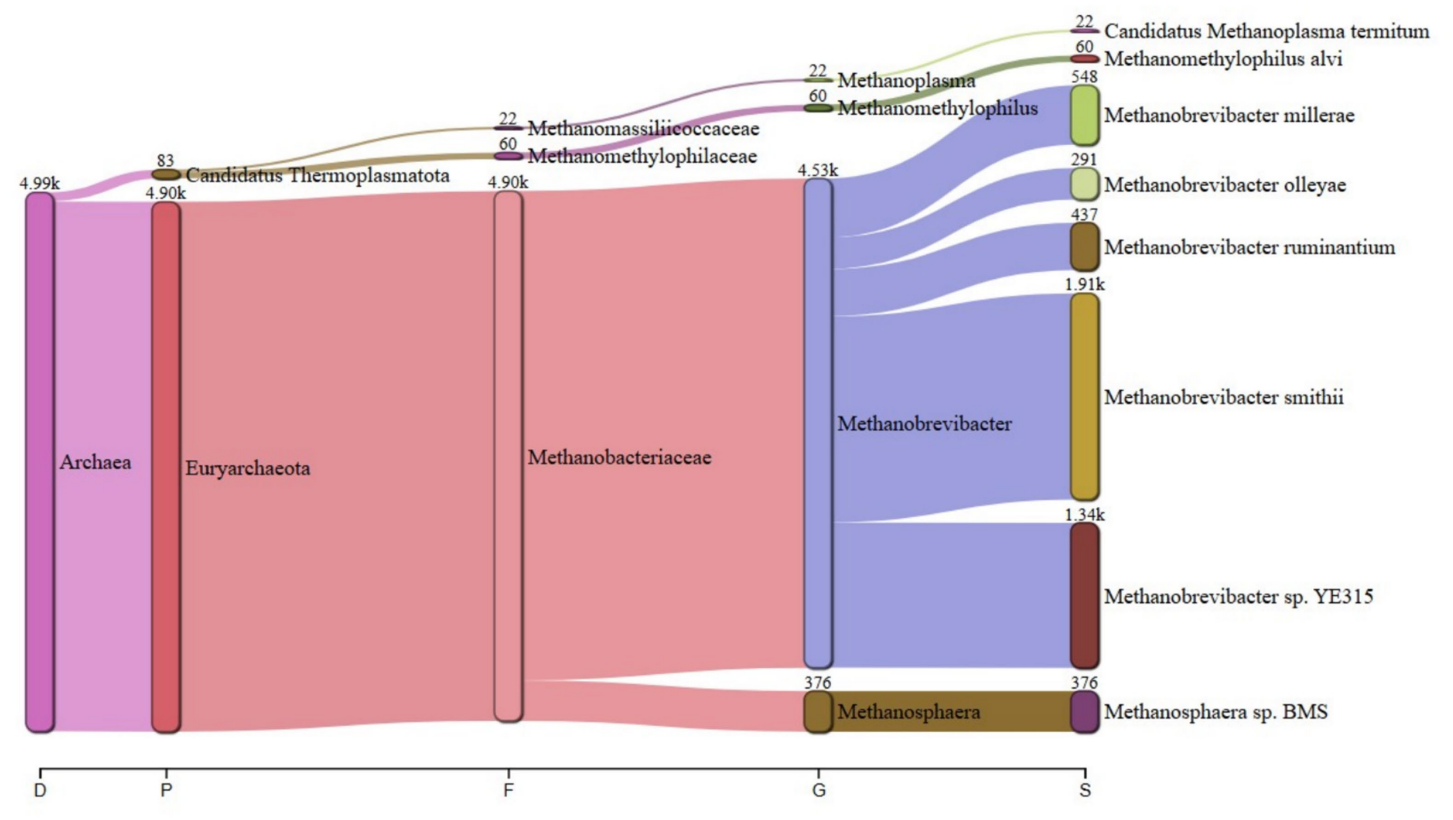
Treatment-specific taxonomic composition of treatment SS:75 dominated by *Methanobrevibacter smithii*.

**Figure 4:**
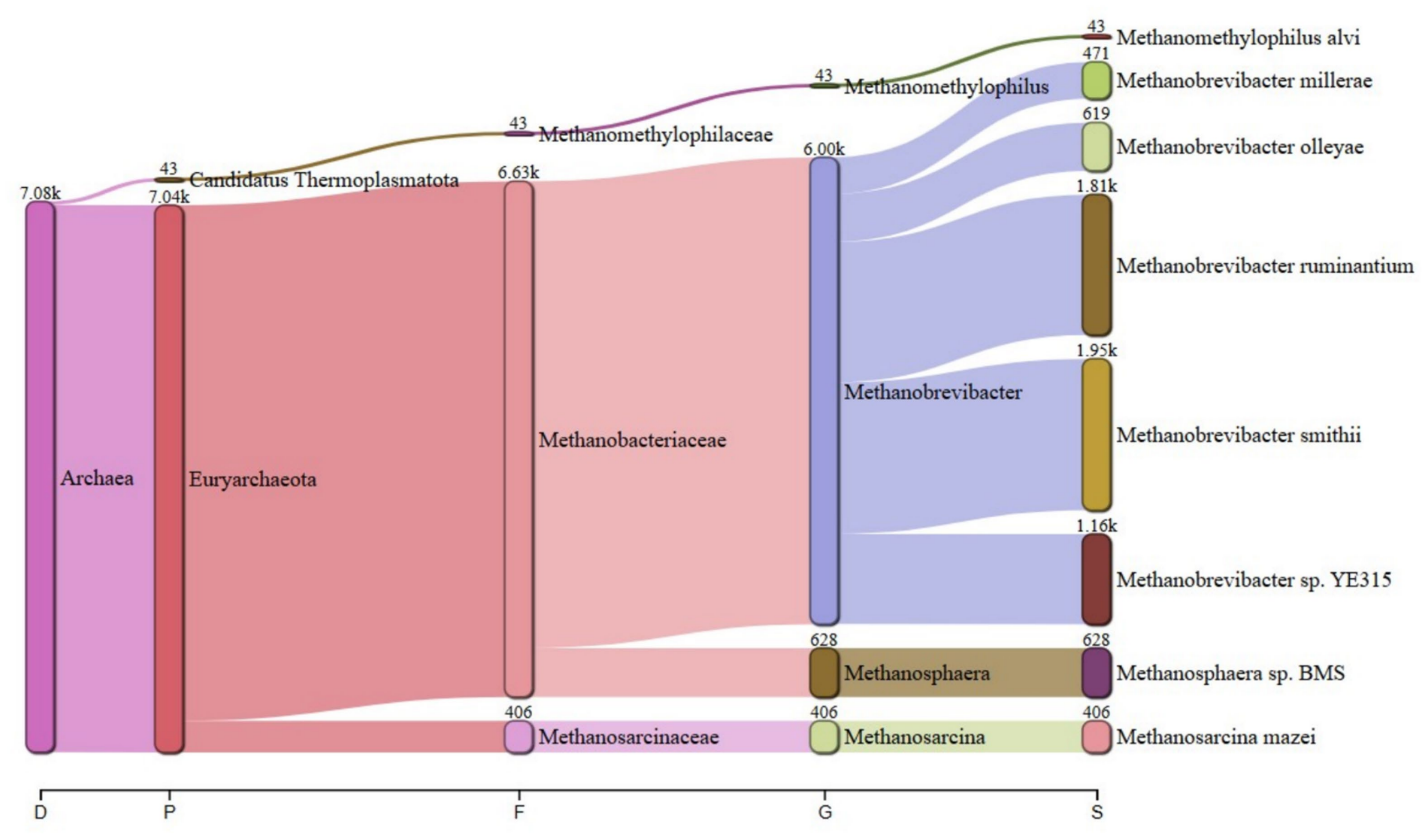
Treatment-specific taxonomic composition of treatment FS:25 with near-exclusive Euryarchaeota.

**Figure 5:**
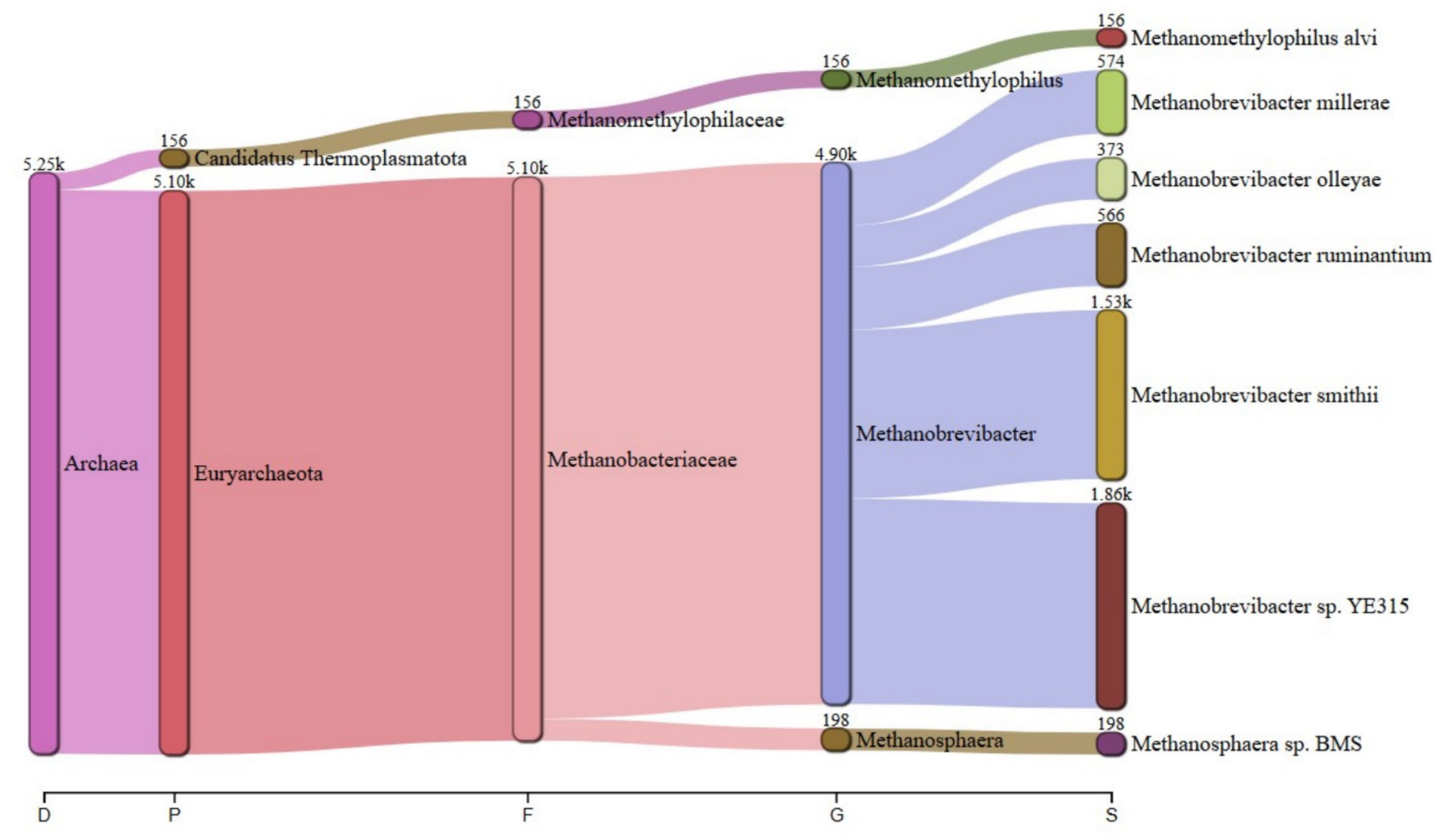
Treatment-specific taxonomic composition of treatment MS:100 dominated by *Methanobrevibacter* sp. *YE315*.

**Figure 6:**
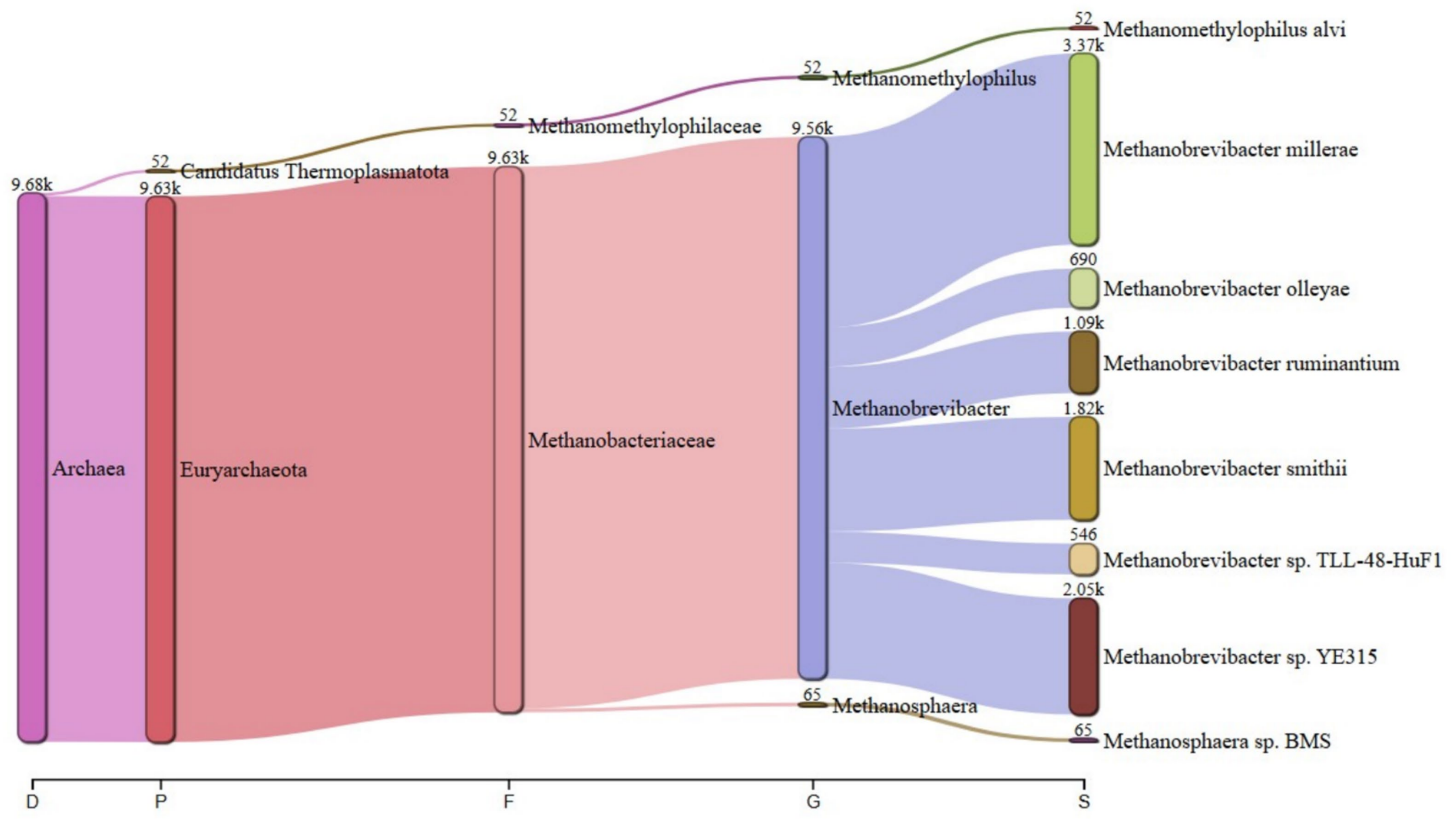
Treatment-specific taxonomic composition of treatment M:100 dominated by *Methanobrevibacter millerae*.

**Figure 7:**
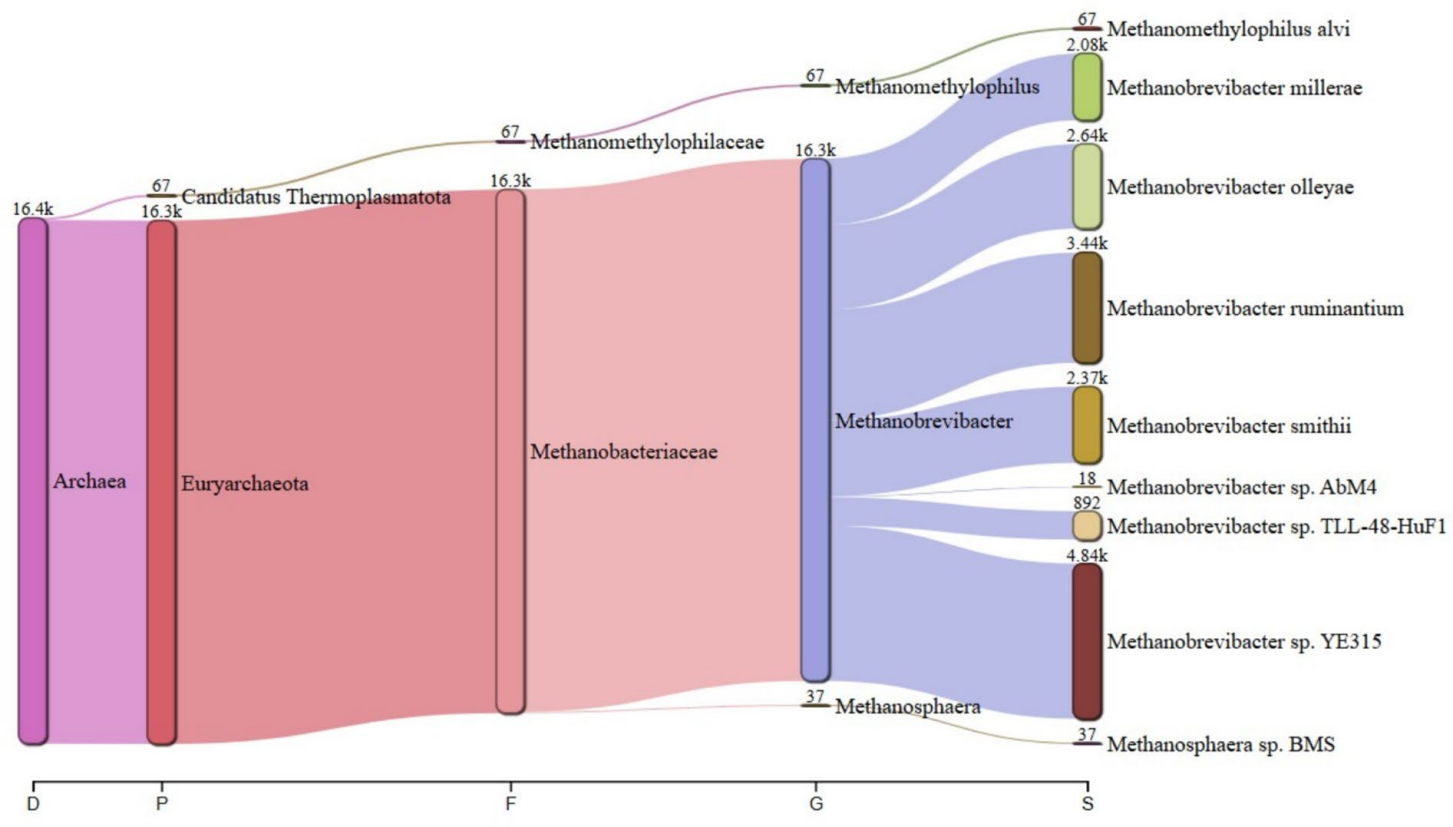
Treatment-specific taxonomic composition of treatment CC dominated by *Methanobrevibacter* sp. *YE315* and *Methanobrevibacter ruminantium*.

The predominant archaeal phyla were Euryarchaeota, averaging 69% and Candidatus Thermoplasmatota, averaging 31% across treatments. Figure 1 summarizes the microbiota abundance at class level and Sankey diagrams (Figures 2–7) illustrate changes in phylum-to-genus abundance. The following trends were observed: Euryarchaeota was the predominant phylum across all treatments, primarily due to the species *Methanobrevibacter*. In FS:25, Euryarchaeota was the near-exclusively dominant archaeal phylum, accounting for 99.4% of total archaeal sequences. The genus *Methanomethylophilus* predominantly accounted for the modest prevalence of Candidatus Thermoplasmatota with PS:50 and MS:100 recording the highest (8–45%) concentration. In accordance with its seldom prevalence, Candidatus Thermoplasmatota was scarcely detected in FS:25 accounting for 0.6% of the sequences.

At the genus level, treatment-specific alterations were evident. Table 2 verifies variations at the genus level: *Methanobrevibacter* (*M. smithii*, *M. ruminantium*), *Methanosarcina* (e.g., *M. mazei*), and *Methanosphaera* (*Methanosphaera* sp. *BMS*) predominated in FS:25, while *Methanomethylophilus* accounted for 55 and 80.5% of the archael sequences in MS:100 and PS:50, respectively, but diminished in Treatments SS:75, M:100 and CC. The genus *Methanobrevibacter* predominated (72.7–86.6%) in M:100, SS:75 and CC diets. *Methanoplasma* was only observed in FS:25 and SS:75, while *Methanosarcina* and *Methanomicrobium* were only observed in FS:25 and CC, respectively. These findings indicate that archaeal community composition varied distinctly across dietary treatments.

**Table 2:** Key taxonomic abundances from Sankey diagrams.

Treatment	*Euryarchaeota* (%)	*Candidatus thermoplasmatota* (%)	Dominant genus (abundance)
PS:50	19.50	80.50	*Methanomethylophilus* (4.70 k)
SS:75	82.00	18.00	*Methanobrevibacter smithii* (18.9 k)
FS:25	99.40	0.60	*Euryarchaeota* genera (6.63 k)
MS:100	45.00	55.00	*Methanomethylophilus* (4.90 k)
M:100	72.70	27.30	*Methanobrevibacter* (25.6 k)
CC	86.60	13.40	*Methanobrevibacter* (106.2 k)

PS:50, 50% plain sorghum diet; SS:75, 75% soaked sorghum diet; MS:100, 100% malted sorghum diet; FS:25, 25% fermented sorghum diet, M:100, 100% maize-based diet; CC, commercial concentrate.

### Alpha diversity (Shannon) across treatments

**Figure 8:**
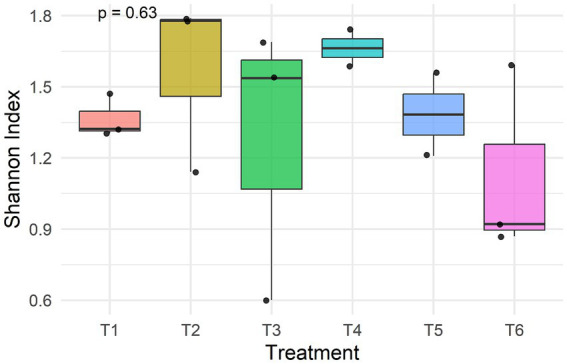
Shannon diversity by treatment, showing variable diversity in the treatments. Treatments: T1-(PS:50)-50% plain sorghum diet; T2-(SS:75)-75% soaked sorghum diet; T3-(FS:25)-25% fermented sorghum diet; T4-(MS:100)-100% malted sorghum diet; T5-(M:100)-100% maize-based diet; T6-(CC-commercial concentrate).

The treatments exhibited numerical differences in Shannon diversity indices as shown in Figure 8. Table 3 displays the Shannon diversity indices by treatment. Table 3 and Figure 8 show that the treatments varied numerically in Shannon diversity indexes. While the commercial diet had the lowest mean Shannon index (1.13 ± 0.40), the MS:100 diet had the highest (1.66 ± 0.11). There were no statistically significant variations in alpha diversity across the six dietary regimens, according to one-way ANOVA (*p* = 0.63).

**Table 3:** Alpha diversity indices (Shannon) by treatment.

Treatment	Shannon index (mean ± SD)
PS:50	1.37 ± 0.10
SS:75	1.57 ± 0.37
FS:25	1.28 ± 0.59
MS:100	1.66 ± 0.11
M:100	1.38 ± 0.25
CC	1.13 ± 0.40

PS:50, 50% plain sorghum diet; SS:75, 75% soaked sorghum diet; MS:100, 100% malted sorghum diet; FS:25, 25% fermented sorghum diet, M:100, 100% maize-based diet; CC, commercial concentrate.

### Beta diversity and treatment clustering (PCoA)

**Figure 9:**
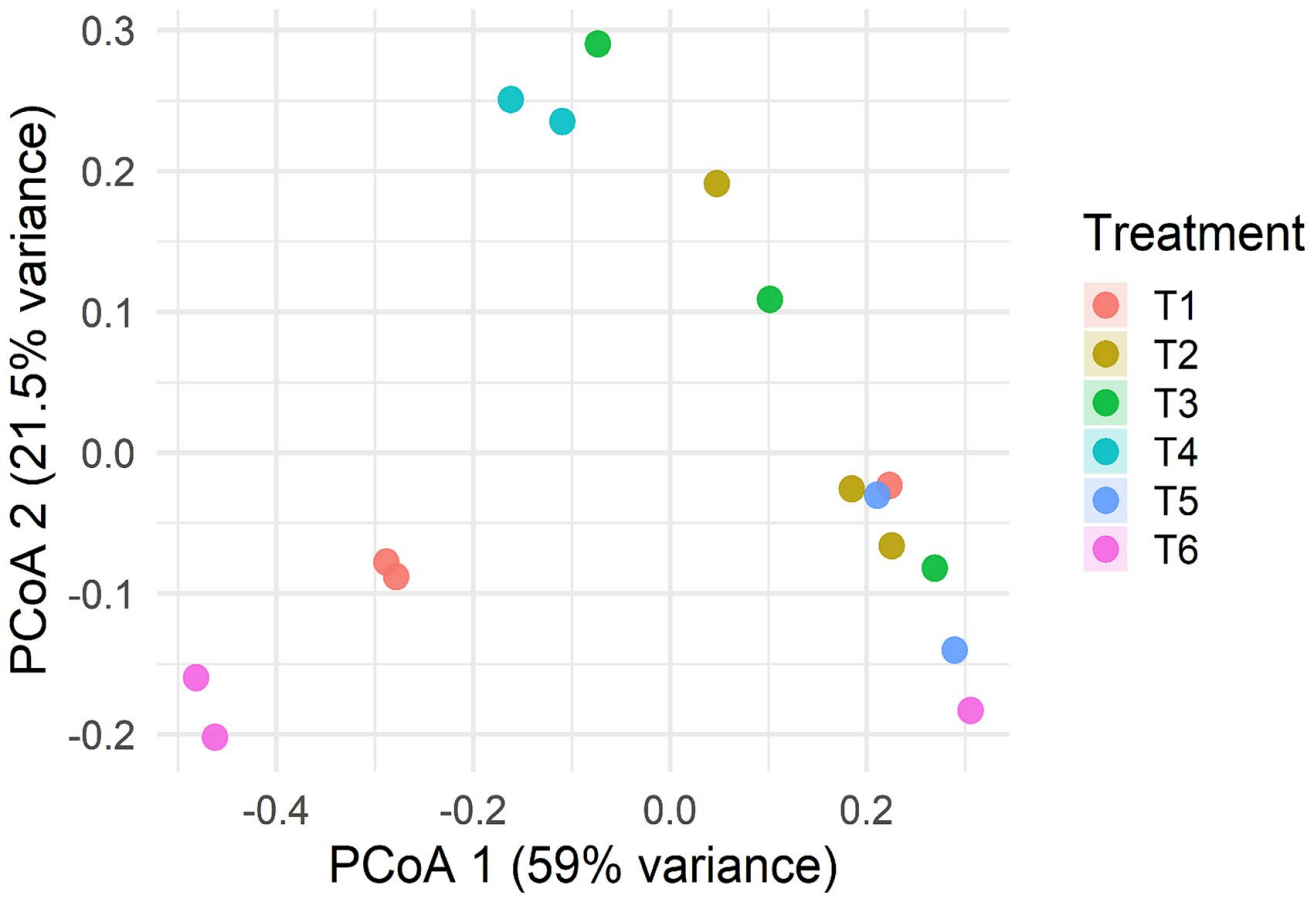
PCoA (Bray–Curtis) of samples, revealing treatment-based clustering of treatments.

Application of principal coordinate analysis (PcoA) to Bray–Curtis distances demonstrated significant grouping based on treatment (Figure 9). PERMANOVA analysis showed that treatment groups explained 78% of taxonomic variance (*R*^2^ = 0.78, *p* = 0.001; Table 4), confirming high cross-tool consistency. Principal coordinate analysis 2 (PCoA2) and principal coordinate analysis 1 (PCoA1) explain 59 and 21.53% of the variance, respectively. The Treatments FS:25 and CC were identified as two distinct outliers. The findings of this study reveal distinct patterns of dominance between *Methanobrevibacter* and *Methanomethylophilus*.

**Table 4:** PERMANOVA results for cross-tool consistency validation (*R*^2^ = 0.78, *p* = 0.001).

	df	SumOfSqs	*R* ^2^	*F*	Pr (>*F*)
Model	1	6.585415	0.779971	106.3458	0.001
Residual	30	1.857737	0.220029	NA	NA
Total	31	8.443152	1	NA	NA

## Discussion

The present study demonstrated that partial or complete substitution of maize with processed sorghum grains altered the ruminal archaeal community composition. These treatment-specific shifts in methanogen community structure suggest that the form of sorghum processing, and its inclusion level, influences the fermentation environment within the rumen in ways that selectively favor distinct methanogenic populations. Understanding these relationships is particularly relevant in the context of southern African smallholder livestock systems, where Mashona goats are valued for their resilience and adaptability, and where dietary interventions using locally available feed resources such as sorghum present a practical avenue for supporting animal production and productivity.

Fasting animals for prolonged periods may induce shifts in rumen microbial community composition. However, research shows that core members of the rumen microbiome, including dominant methanogenic archaea, maintain remarkable temporal stability and functional redundancy (Lima et al., 2024). Work by Kim et al. (2018) found that the numbers of key microorganisms in the rumen, such as protozoa, methanogenic archaea, and anaerobic fungi, did not differ between control and fasted animal. The study found the archaeal community to respond differently to every dietary treatment, concurring with earlier research (Henderson et al., 2015; Mao et al., 2024). This was an indication that variations in microbial compositions were largely due to feed rather than the host animal. Characterization of the rumen archaeal community revealed a clear dominance of the phylum Euryarchaeota. Within the same phylum, *Methanobrevibacter* was identified as the predominant genus (Chaucheyras-Durand and Ossa, 2014), accounting for 55–92% of archaeal sequences across the experimental diets. This observation is congruent with findings by Wei et al. (2024) who observed dominance of Euryarchaeota (>99.8%) and its genus *Methanobrevibacter* (>99.4%) in sheep on a hydroethanolic walnut green husk extract diet. *Methanobrevibacter* species are well-established hydrogenotrophic methanogens, that convert hydrogen and carbon dioxide into methane (Króliczewska et al., 2023). This agrees with several studies (Kumar et al., 2015; Li et al., 2021; Rabee et al., 2024; Wang et al., 2017) that reported that more than 90% of sequences of rumen archaea analysed from public databases are affiliated with the methane producing genera *Methanobrevibacter*. Candidatus Thermoplasmatota, primarily represented by the genus *Methanomethylophilus* was found in moderate to low abundance across all treatments. This finding was similar with observations by Fliegerova et al. (2021) who noted the genus in low (3.3%) abundance in goats fed a high concentrate diet. Conversely, research by Li et al. (2023) demonstrated that *Methanomethylophilus* represented a major methanogenic group in cashmere goats consuming alfalfa hay.

*Methanomethylophilus* utilize methylated compounds like methanol, methylamines, and methyl sulfides for methane production (Mackie et al., 2024) unlike hydrogen and carbon dioxide used by *Methanobrevibacter* (Króliczewska et al., 2023). The dominance of *Methanomethylophilus* in PS:50 and MS:100 diets may imply a shift in the hydrogen sink mechanisms and substrate availability within the rumen, driven by dietary factors. Earlier work by Hassan F. U. et al. (2020) and Hassan S. et al. (2020) suggested that the use of tannins may reduce fiber digestion thereby decreasing hydrogen availability. This can modify the rumen microbiome, promoting the proliferation of methanogens such as *Methanomethylophilus* that are capable of utilizing alternative, methylated substrates (Hassan F. U. et al., 2020; Hassan S. et al., 2020). Dietary fiber and nutritional content of feed significantly influences archaeal community composition and diversity (Li et al., 2025). Therefore, the observed differences in *Methanomethylophilus* abundance may stem from variations in fiber, fat and nutrient levels across diets, rather than solely from the sorghum processing methods used. The higher inclusion rates of unprocessed sorghum (50%) in the PS:50 diet and 100% in the MS:100 diet, along with the malting method used for MS:100, may have also contributed to the increased availability of methyl-donating compounds. This may have created an environment that favored methylotrophic methanogens like *Methanomethylophilus*, allowing them to thrive due to enhanced substrate availability. Additionally, differences in crude protein and ether extract content between diets may have influenced archaeal populations, as protein levels affect ruminal ammonia nitrogen availability (Ohene-Adjei et al., 2008). Tannins are also known to exhibit antimicrobial activity to microbes which produce the hydrogen required for methane production (Lileikis et al., 2023). Given that the red sorghum variety used in the study contains varying levels of condensed tannins (Dykes and Rooney, 2006), we hypothesize that tannins may have also contributed to the observed archaeal shifts. This reduction in hydrogen could theoretically favor methylotrophic methanogens like *Methanomethylophilus*, which utilize methylated compounds rather than hydrogen for methanogenesis (Mackie et al., 2024).

The near-total dominance of Euryarchaeota in the FS:25 was rather unexpected as the diet was formulated from fermented sorghum at 25% sorghum inclusion. Previous studies reported a decline in the abundance of *Methanobrevibacter* in animals supplemented with tannins (Hassan F. U. et al., 2020; Hassan S. et al., 2020). Another study (Xie et al., 2023) also noted a decline in the proportional representation of methanogenic archaea in steers fed sorghum grain. This extreme shift observed in the FS:25 diet suggests that the specific formulation or processing of sorghum, coupled with the low sorghum inclusion (25%), reduced the tannin content and that may have potentially established optimal conditions for the proliferation of *Methanobrevibacter*. The generation of a micro-niche in FS:25 may have resulted in suppression of other species. A decline in the abundance of one methanogen may result in the thriving of another (Cieslak et al., 2013).

Diet is a primary driver of the rumen microbial community’s activity, richness, and diversity (Palmonari et al., 2024). The results of the current study demonstrated that the archaeal community’s Shannon diversity indices varied numerically across treatments. Studies have shown that refined diets can diminish microbial diversity due to a limited spectrum of fermentable substrates. The commercial concentrate had the numerically lowest diversity index, which is consistent with this pattern (Henderson et al., 2015). The low microbiota diversity in this diet could be attributed to multiple interacting factors including its refined formulation and specific NDF/ADF content. While refined diets are known to reduce microbial diversity due to a limited range of substrates available for fermentation (Henderson et al., 2015), the NDF/ADF ratio may have been a key determinant of archaeal diversity. Research by Bai et al. (2022) demonstrated that increasing dietary NDF ratios significantly altered archaeal community composition and alpha diversity. Therefore, the observed low diversity in the commercial concentrate (CC) likely reflects the combined influence of its refined nature and specific fiber composition. Earlier work (Belanche et al., 2012) observed a complex microbial community in cows fed diets rich in fiber as an adaptation strategy to the fibrous diet. Diets high in starch-rich concentrates are associated with decreased ruminal pH and subsequent acidosis, which negatively affects rumen microbiota diversity and richness (Neubauer et al., 2020). High-grain diets are known to decrease the richness, evenness, and overall ruminal microbial diversity (Khafipour et al., 2016). Conversely, a study on dairy calves found that those fed a milk and concentrate diet had increased proportions of methanogenic archaea and carbohydrate-degrading bacteria compared to those fed milk exclusively at 28 days of age (Dias et al., 2017).

Interestingly, the FS:25 diet formulated from 25% fermented sorghum, also exhibited also exhibited numerically reduced diversity which was an unexpected outcome, given the observed high evenness among Euryarchaeota genera in this treatment. The diet was expected to exhibit higher diversity as an optimal inclusion rate of 25–50% sorghum in goat diets has been established as safe and effective (Saka et al., 2020). The overwhelming dominance of phylum Euryarchaeota, however, significantly reduced overall diversity. Similarly, AlZahal et al. (2017) observed a reduction in diversity driven by low species richness and increased dominance of certain species in dairy cattle fed a high grain diet. While some microorganisms thrive on the increased substrate and provided by certain diets, others suffer due to the reduced fiber availability and the negative effects of lower pH or elevated gut metabolites (Khafipour et al., 2016). The current study’s results might therefore suggest that while the energy source is abundant, the type of available substrate might be narrow, limiting the overall archaeal diversity. Fermentation is known to modify the structural properties of starch, increasing the availability of fermentable sugars (Wei et al., 2022). Increased fermentability might typically support a richer microbial community. However, rapid and extensive fermentation of readily available sugars could result in a less diverse, more specialized community, dominated by fast-growing, highly efficient archaea that exploit these specific substrates. Rumen archaea are also known to be less diverse than rumen bacteria because of their limited substrate range (Henderson et al., 2015; Króliczewska et al., 2023).

In stark contrast MS:100 formulated from malted sorghum at 100% inclusion displayed the highest diversity. This finding contradicts work by Xie et al. (2023) who noted decreased rumen and fecal diversity in steers fed sorghum grain. In agreement with findings from this study, Gao et al. (2023) observed a stable rumen environment in sheep fed sorghum silage, contrasting with the significant fluctuations in bacterial abundance and function observed in sheep fed corn silage. The high diversity observed in MS:100 may reflect the combined effects of malting-induced changes in substrate availability and the specific nutritional profile of this diet. Malting alters the nutritional composition of sorghum grain, increasing the availability of soluble sugars, amino acids, and vitamins, while potentially modifying the fiber structure and NDF/ADF content (Oseguera-Toledo et al., 2020). The malting process can also introduce beneficial microorganisms, including lactic acid bacteria (LAB) and yeasts (Byakika et al., 2021) which can produce metabolites that serve as substrates or growth factors for archaea. This enhanced nutrient availability could support a more varied microbial population, including a wider range of archaeal species. The specific representation of 45% Euryarchaeota and 55% Candidatus Thermoplasmatota in this diet suggests a healthy balance between hydrogenotrophic and methylotrophic methanogens.

Among all treatments, diet SS:75 had the second-highest mean Shannon diversity index. The moderate to low abundance of Candidatus Thermoplasmatota, represented by *Methanomethylophilus*, suggests a relatively consistent role for methylotrophic methanogenesis in this treatment. Its influence however, may be more pronounced in the PS:50 and MS:100 diets. This relatively higher diversity could be attributed to both the soaking process, which was found to increase the proportion of fiber content (Usman and Bolade, 2017), and the resulting NDF/ADF profile of the diet. The interplay between processing method and nutritional composition is particularly relevant here, as soaking-induced changes in fiber content may have created conditions conducive to a broader archaeal community. Feeds with increased fiber tend to promote a broader range of microbial niches (Henderson and Duan, 2024) by subtly altering the physical or chemical properties of the sorghum without introducing highly selective pressures.

Results show that the diets FS:25 and the commercial concentrate were the two distinct outliers. Although both diets allowed archaea to flourish, the Shannon diversity indices were numerically lowest and the Candidatus Thermoplasmatota phylum was under-represented in both. A low alpha diversity in a microbial community is not always a disadvantage (Shade, 2017). A less diverse but highly efficient microbial community may be optimal for specific feed substrates. However, reduced diversity can also indicate a less resilient ecosystem, potentially more susceptible to disruptions (Brunn et al., 2024). While the findings revealed treatment-specific shifts in ruminal archaeal community composition across the sorghum-based and maize-based diets, the study did not include direct measurement of enteric methane emissions, which would be necessary to confirm whether the observed changes in methanogen community structure translated into quantifiable differences in methane output. Additionally, rumen physicochemical and metabolite data, including rumen pH, volatile fatty acid profiles, and hydrogen concentrations, would have provided mechanistic context for the observed shifts in archaeal community composition.

## Conclusion

Diet influences rumen ecology by shaping microbial structure, fermentation patterns, and ecosystem stability. Shifts in microbial structure can lead to changes in metabolic outputs, affecting protein synthesis, nutrient absorption and overall fermentation efficiency. The rumen archaeal community consistently responded uniquely to each dietary treatment, highlighting the sensitivity of the methanogenic archaea to dietary shifts. These responses reflect the combined influence of both sorghum processing methods and differences in nutritional composition (NDF, ADF, crude protein, and ether extract) in the experimental diets. Across all diets, the phylum Euryarchaeota exhibited clear dominance, with *Methanobrevibacter* identified as the predominant genus. The MS:100 diet recorded the highest numerical archaeal diversity, characterized by a near-equal representation of *Methanobrevibacter* and Candidatus Thermoplasmatota. This co-occurrence suggests the simultaneous activity of hydrogenotrophic and methylotrophic methanogenic pathways, with both hydrogen-utilizing and methyl-compound-utilizing methanogens contributing to the archaeal community under this dietary treatment. This diversity in the methanogenic community reflects an integrated rumen ecosystem function, where various fermentation pathways produce hydrogen and methylated substrates, supporting multiple methanogenic niches. The low species diversity and dominance of Euryarchaeota in FS:25 and the commercial concentrate suggest the generation of micro-niches in these dietsFuture research need to prioritize quantifying *in vivo* methane emissions of the sorghum-based diets used in the present to confirm their methane mitigation potential in live animals, thereby solidifying its status as an optimal dietary intervention. Additionally, longitudinal studies investigating rumen microbiome changes during dietary transitions would offer valuable insights into how sorghum-based diets influence rumen ecology and methane production. Complementary functional metabolomics approaches to analyze volatile fatty acid profiles and fermentation pathways would provide deeper understanding of these dietary effects.

## Data Availability

The data presented in this study are publicly available. The data can be found here: https://doi.org/10.6084/m9.figshare.30813803.
